# Study on Microstructure and Mechanical Properties of WC-10Ni_3_Al Cemented Carbide Prepared by Different Ball-Milling Suspension

**DOI:** 10.3390/ma12142224

**Published:** 2019-07-10

**Authors:** Minai Zhang, Zhun Cheng, Jingmao Li, Shengguan Qu, Xiaoqiang Li

**Affiliations:** 1 Guangdong Key Laboratory for Advanced Metallic Materials Fabrication and Forming, National Engineering Research Center of Near-Net-Shape Forming for Metallic Materials, South China University of Technology, Guangzhou 510640, China; 2 Department of Materials Science and Engineering, University of California, Irvine, CA 92697, USA

**Keywords:** WC-10Ni_3_Al, oxygen, ball-milling suspension, α-Al_2_O_3_, η-phase

## Abstract

In this paper, WC-10Ni_3_Al cemented carbides were prepared by the powder metallurgy method, and the effects of ball-milling powders with two different organic solvents on the microstructure and mechanical properties of cemented carbides were studied. We show that the oxygen in the organic solvent can be absorbed into the mixed powders by ball-milling when ethanol (CH_3_CH_2_OH) is used as a ball-milling suspension. This oxygen leads to the formation of α-Al_2_O_3_ during sintering, which improves the fracture toughness, due to crack deflection and bridging, while the formation of η-phase (Ni_3_W_3_C) inhibits the grain growth and increases the hardness. Alternatively, samples milled using cyclohexane (C_6_H_12_) showed grain growth during processing, which led to a decrease in hardness. Therefore, the increase of oxygen content from using organic solvents during milling improves the properties of WC-Ni_3_Al composites. The growth of WC grains can be inhibited and the hardness can be improved without loss of toughness by self-generating α-Al_2_O_3_ and η-phase (Ni_3_W_3_C).

## 1. Introduction

WC-Co cemented carbide is widely used in cutting, mining, and manufacturing fields, due to its excellent combination of hardness and toughness [1,2,3]. In WC-Co cemented carbide, however, WC grain size strongly affects its mechanical properties. Carbide grain growth inhibitors (GGI), such as VC, NbC, and Cr_3_C_2_, are usually added to WC-Co systems to restrain the growth of WC grains and improve the hardness and strength [4,5,6,7,8]. However, the GGI content must be restricted to small amounts (less than 1%) [5] because too much will lead to GGI precipitation at the grain boundary and reduce the fracture toughness. In addition, high-temperature performance and corrosion resistance of WC-Co are not good [9]. The shortage of Co resources and its toxicity to the human body further limit its application [10].

Ni_3_Al-based intermetallics are considered a promising alternative to Co because of their good wettability with WC, high-temperature properties and corrosion resistance [11]. Li et al. [12] prepared WC-10 wt.% Ni_3_Al cemented carbide with excellent mechanical properties (hardness HV_10_ is 17.76 GPA, transverse rupture strength (TRS) is 2092 MPa, and fracture toughness is 21.56 MPa·m^1/2^) by using spark plasma sintering (SPS). Long et al. [13] reported that WC-40 vol.% (Ni_3_Al-B) composites could be prepared by the pre-alloy reaction of pure nickel powder, pure aluminum powder and WC powder followed by sintering. The hardness of these composites was 9.7 GPa, the bending strength was 1800 MPa, and the fracture toughness was 18 MPa·m^1/2^. Liang et al. [14] studied the cutting performances of WC-10 wt.% Ni_3_Al compared with that of WC-8 wt.% Co. It is demonstrated that WC-10Ni_3_Al has higher crater and flank wear resistance than WC-8 wt.% Co, which is attributed to the synergistic mechanism of chemical inertness and superior hardness induced by Ni_3_Al binder at high temperature. Liu et al. [15] also found that WC-8 wt.% Ni_3_Al cemented carbide had better wear resistance than WC-8 wt.% Co cemented carbide, because WC-8 wt.% Ni_3_Al cemented carbide has higher hardness and Ni_3_Al induced subsurface crack resistance. The oxidation resistance of WC-Ni_3_Al alloy was improved compared with that of cemented carbide WC-Co, which is due to the formation of a dense oxide layer on the WC-Ni_3_Al substrate, and the good combination of the oxide layer with the substrate [9].

These studies were carried out on WC-Ni_3_Al cemented carbides using the powder metallurgy (PM) method. Thus, the powder preparation process is crucial, especially the control of oxygen content. However, the powder will inevitably be oxidized in the PM process. The influence of different ball-milling suspension fluids on the oxygen content of WC-Ni_3_Al cemented carbide has not been reported. In this paper, we will study the effect of two different ball-milling suspensions (ethanol and cyclohexane) on the oxygen content, microstructure, and mechanical properties of WC-Ni_3_Al cemented carbide. 

## 2. Materials and Methods 

First, Ni_3_Al alloy powder with a composition of Ni-8.0Al-7.7Cr-1.43Mo-0.008B (wt.%) was prepared by planetary ball-milling (QM-2SP20, Nanjing NanDa Instrument Plant, Nanjing, China) using WC balls in stainless-steel jars with a rotation speed of 226 rpm and a ball-to-powder weight ratio of 10:1 for 50 h under argon atmosphere. After sieving with 100 mesh, Ni_3_Al powder was blended with WC powder (0.8 μm, 99.9%, Jinglu Lt. Co., Xiamen, China) in a composition of WC-10 wt.% Ni_3_Al on a V-type mixer. In order to disperse the powder more evenly, the blended powders were then mixed also by ball-milling with a ball-to-powder weight ratio of 4:1 and a rotation speed of 180 rpm for 24 h. Cyclohexane (C_6_H_12_) or ethanol (CH_3_CH_2_OH) were used as milling suspensions, respectively. The mixed powders were sieved after vacuum drying to avoid agglomeration. Before sintering, 26 g mixed powders were packed in graphite die with a size of ϕ20 mm. Finally, bulk WC-10%Ni_3_Al cemented carbides were obtained by SPS (Sumitomo Coal Mining Co., Ltd., Ichikawa, Japan) at 1400 °C for 5 min.

The two kinds of samples were characterized by X-ray diffraction (XRD, D8 Advance, Bruker Co., Hamburg, Germany), scanning electron microscope (SEM, Quanta3D, FEI, Hillsboro, OR, USA), and transmission electron microscope (TEM, JEOL2100F, JEOL, Tokyo, Japan). The oxygen content of powder before and after mixing, and in the bulk state were analyzed by a TC600 N/O Analyzer (Leco, St Joseph, MI, USA). The hardness of the specimens were measured using the Vickers hardness method, with each specimen being measured at least five times. The fracture toughness was measured by the indentation method and calculated by Shetty’s equation [16].

## 3. Results and Discussion

**Figure 1:**
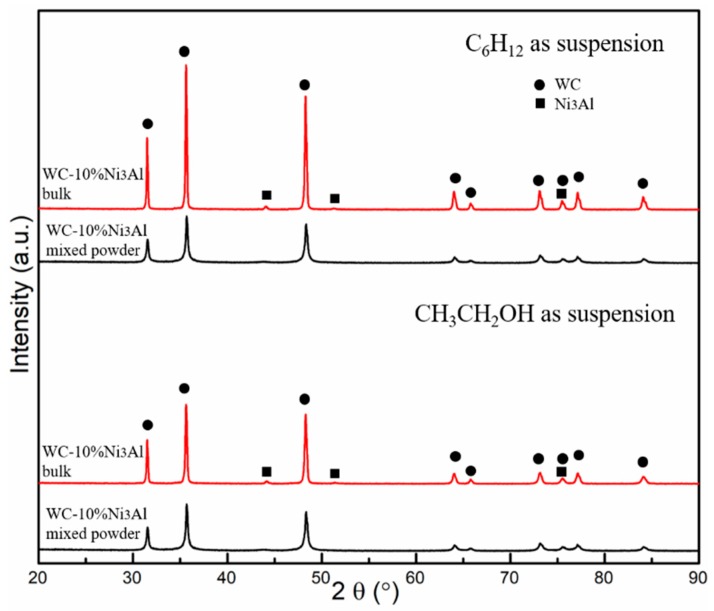
X-ray diffraction (XRD) patterns of WC-10Ni_3_Al mixed powder and bulk using different ball-milling suspensions.

Figure 1 shows the XRD patterns of WC-10Ni_3_Al composite powders and sintered bulk materials using different ball-milling suspensions. It can be found that the XRD patterns of using two kinds of ball-milling suspensions are the same. The characteristic peaks of the mixed powder were wider and shorter, while the characteristic peaks of the sintered block were narrower and higher, which was due to the grain growth during sintering. In the WC-Co system, according to the Ostwald ripening [17], WC grain growth can be described as the dissolution of smaller WC particles into binder Co phase during heating. The dissolved WC particles re-precipitated on the surface of the larger undissolved WC particles, which promoted the growth of WC grains. Since both Co and Ni_3_Al can dissolve a certain amount of WC near the sintering temperature [18], the same mechanism should occur in WC-Ni_3_Al system. In addition, the results show that the sintered samples are both composed of WC and Ni_3_Al.

**Figure 2:**
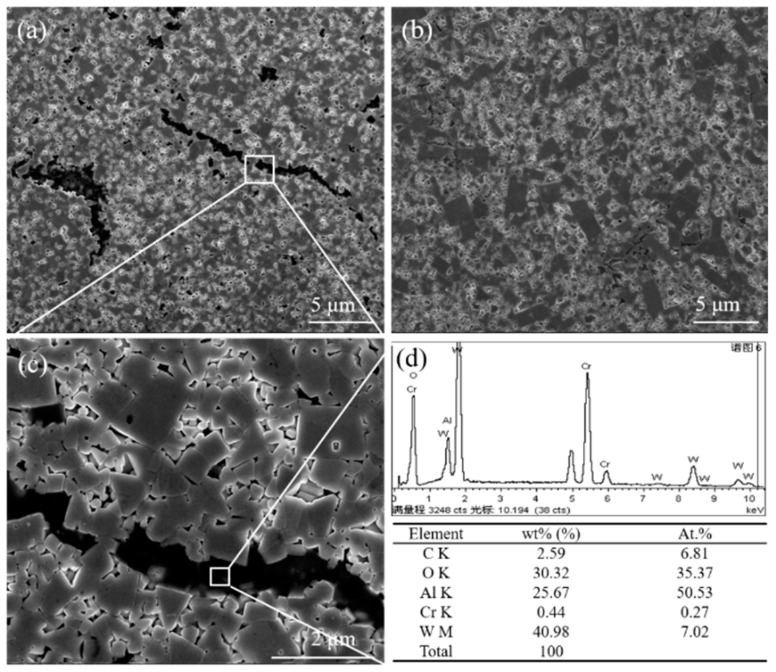
Microstructure of WC-10Ni_3_Al bulk materials using different ball-milling suspensions, (**a**) CH_3_CH_2_OH, (**b**) C_6_H_12_, (**c**) the details from the box in (**a**), (**d**) X-ray spectroscopy (EDS) for the black phase.

Figure 2 shows the microstructure of WC-10Ni_3_Al bulk materials using different ball-milling suspensions. There are noticeable dark regions in the micrographs when CH_3_CH_2_OH was used as milling suspension. Figure 2c shows a more detailed micrograph of the boxed region in Figure 2a, while Figure 2d is the corresponding energy dispersion X-ray spectroscopy (EDS, X-act one, Oxford, Oxford, UK) analysis. The results show that there was a significant amount of O in the black phase, where the ratio of Al and O atoms was basically in agreement with that of Al_2_O_3_. We can preliminarily determine that this phase is Al_2_O_3_. The reason why the existence of Al_2_O_3_ was not observed in the XRD pattern may be due to the high X-ray absorption coefficient of Al_2_O_3_ [19]. Additionally, the Al_2_O_3_ content in the samples was likely insufficient to be detected by XRD. While in Figure 2b, there was no black phase distribution, which means that no Al_2_O_3_ was produced in WC-Ni_3_Al cemented carbide while using C_6_H_12_ as the milling suspension. Therefore, in the mixing process of wet milling, the adsorption of oxygen in the mixed powder is strongly influenced by the organic solvent in the ball-milling suspension. In addition, it should be noted that in the samples containing Al_2_O_3_, the WC grains were fine and uniform, while using C_6_H_12_, some grains grew abnormally as plate-like grains.

**Figure 3:**
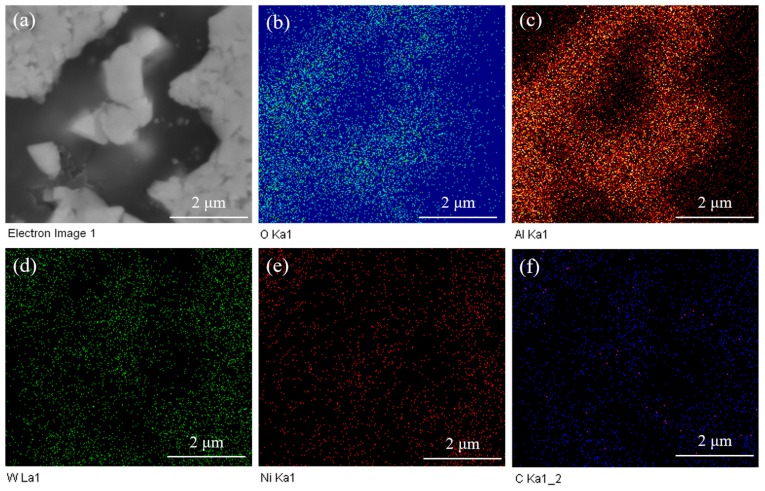
EDS maps of a sintered WC-10Ni_3_Al composite using CH_3_CH_2_OH as ball-milling suspension, (**a**) SEM image; (**b**) O, (**c**) Al, (**d**) W, (**e**) Ni, (**f**) C.

Figure 3 shows the EDS mapping of WC-10Ni_3_Al bulk materials using CH_3_CH_2_OH as ball-milling suspension. The different colors represent the distribution of different elements. The black phase in the electron image was the region where Al and O atoms congregate, W atoms were the opposite, and other elements were evenly distributed. Our results indicate that the black phase is formed by the aggregation of oxide.

**Figure 4:**
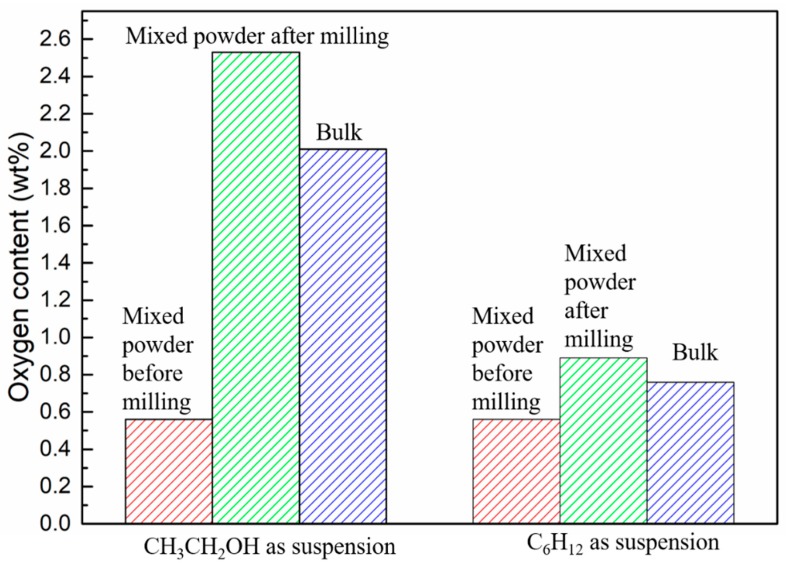
Oxygen content of WC-Ni_3_Al powders before and after milling and in sintered bulk composites.

In order to investigate the source of oxygen, the oxygen contents of WC-10Ni_3_Al composites before and after ball-milling, and bulk materials were measured. As shown in Figure 4, there was a certain oxygen content (about 0.5%) in the powder before ball-milling, which should be the oxygen absorbed by the powder in the air or dissolved in the gap between the powder particles. The oxygen content of mixed powder after ball-milling with CH_3_CH_2_OH was 2.53%, while the oxygen content of mixed powder after ball-milling with C_6_H_12_ was not significantly increased from 0.56 to 0.89. It is suggested that oxygen atoms in organic solvent CH_3_CH_2_OH can be adsorbed on the surface of the powder or dissolved in the interspace of the powder particles, resulting in the increase of oxygen content. Although there were no O atoms in C_6_H_12_ ball-milling suspension, there is a little air in the ball-milling jar. The adsorption of oxygen on the surface of the powder increases with the increase of the specific surface area of the wet-milling powder. In addition, the ball-milling process inevitably leads to the reduction of particle size, and due to surface energy and core-shell constellation, the oxygen content of the powder increases. Therefore, the oxygen in the CH_3_CH_2_OH ball-milled powder mainly comes from the adsorption and dissolution of O atoms in the organic solvent. In addition, we can see that the oxygen content of the bulk materials decreases after sintering, which indicates that oxygen may participate in the gas reaction and lead to the loss of oxygen content during sintering.

**Figure 5:**
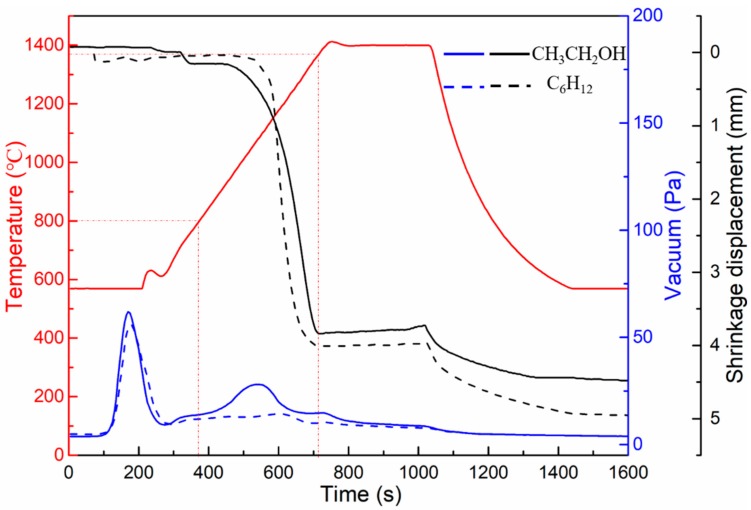
Densification curves for specimens sintered at 1400 °C using different ball-milling suspensions.

Figure 5 shows the sintering curves of WC-10Ni_3_Al composites at 1400 °C for 5 min (including the curves of displacement, vacuum, and temperature with time) in different ball-milling suspensions. Since the temperature range measured by the infrared thermometer was above 570 °C, the temperature appears to be 570 °C before 4 min, and in fact, the temperature rises from room temperature to this temperature during this period. There was a decrease in the vacuum level in both mixed powders at 570 °C, which has been shown to be the gasification process of water and other volatile substances in the powders [20]. In addition, it can be found that from 800 °C, the vacuum level of the mixed powder using CH_3_CH_2_OH as the ball-milling suspension decreases with increasing temperature, while the change was not obvious in the mixed powder using C_6_H_12_ as the ball-milling suspension. This means that gas is produced at this stage in the CH_3_CH_2_OH milled powders, but not in the C_6_H_12_ powders. On the basis of the decrease of oxygen content after sintering and the investigation of the Ellingham Diagram [21], it is suggested that the oxygen adsorbed on the surface of the powder and the dissolved oxygen in the gap between the powder particles may react with carbon to form CO, because the formation free energy of CO is lower than that of CO_2_ and the formation temperature of CO is within this temperature range. This explains why the oxygen content of the bulk materials in Figure 4 is lower than that of the powders. Even though carbon and oxygen were lost from WC-10Ni_3_Al as CO gas, the oxygen content in the bulk material milled with CH_3_CH_2_OH was as high as 2.01%. This remaining oxygen should be mainly in the form of Al_2_O_3_ oxide, because according to the Ellingham Diagram [21], the free energy of oxide formation of W, Ni and Cr are high. In addition, according to the shrinkage and displacement of the samples, the samples milled using CH_3_CH_2_OH start densification at ~800 °C, which is consistent with the temperature at which CO gas is produced. While the samples milled using C_6_H_12_ start densification at ~1000 °C. Both samples were densified at 1350 °C, so the sintering temperature of 1400 °C was reasonable.

**Figure 6:**
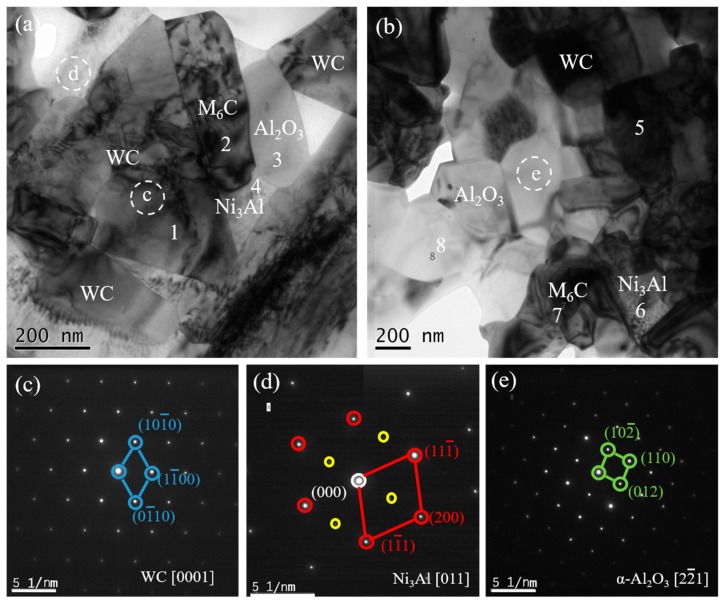
TEM images of the WC-10Ni_3_Al specimen using CH_3_CH_2_OH and selected area electron diffraction patterns obtained from the different phases. (**a**) local area a; (**b**) local area b; (**c**–**e**) selected area diffraction pattern (SADP) corresponding to the white dotted circles.

To further confirm the phase composition of WC-10Ni_3_Al composites, Figure 6 shows TEM images of bulk using CH_3_CH_2_OH as the ball-milling suspension. Figure 6c,d is the selected area diffraction pattern (SADP) corresponding to the white dotted circles in Figure 6a,b. After analysis, it can be identified that the black phases with triangular and quadrilateral shape are the WC matrix, and the Ni_3_Al phases are distributed among the WC grains. It is worth mentioning that the SADP of Ni_3_Al phases has a standard superlattice structure, as shown in Figure 6d. The white phases in the micrographs are polycrystalline α-Al_2_O_3_. In addition, EDS was used to analyze the elemental composition of the numbered spots in Figure 6a,b, as summarized in Table 1. According to the element atomic ratio, spots 1 and 5 are the WC phase, spots 4 and 6 are the Ni_3_Al phase, and spots 3 and 8 are the Al_2_O_3_ phase. It has been determined that the gas produced during the reaction between oxygen and carbon leads to the carbon deficiency of the matrix. On the other hand, the production of oxide Al_2_O_3_ must be accompanied by the change of the composition of Ni_3_Al phase and the formation of a η-phase (Ni_3_W_3_C) to maintain the carbon balance. According to EDS results, a small amount of η-phase (Ni_3_W_3_C) was found around the α-Al_2_O_3_ phase (spots 2 and 7). The results show that the organic solvent has a great influence on the oxygen content of the WC-Ni_3_Al composite, and the absorbed or dissolved oxygen can change the phase composition of the WC-Ni_3_Al composite.

**Figure 7:**
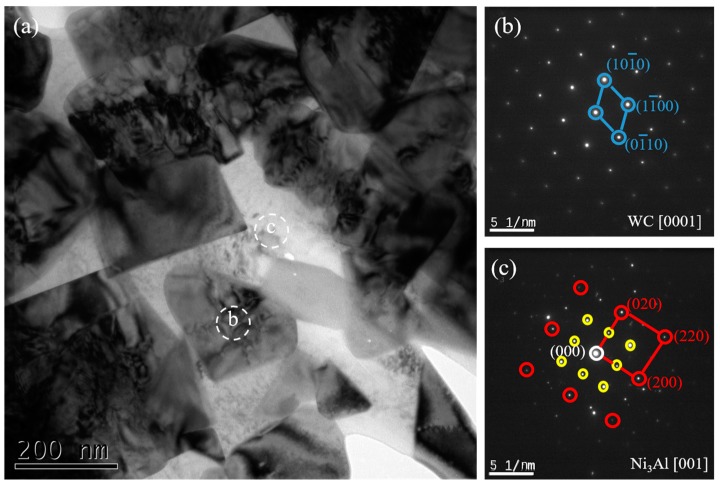
TEM image of the WC-10Ni_3_Al specimen using C_6_H_12_ and selected area electron diffraction patterns obtained from the different phases. (**a**) local area; (**b**,**c**) selected area diffraction pattern (SADP) corresponding to the white dotted circles.

Figure 7 shows TEM images of the WC-10Ni_3_Al samples milled by C_6_H_12_ and the SADP of each phase. Only two phases exist in this sample, WC and Ni_3_Al. 

**Figure 8:**
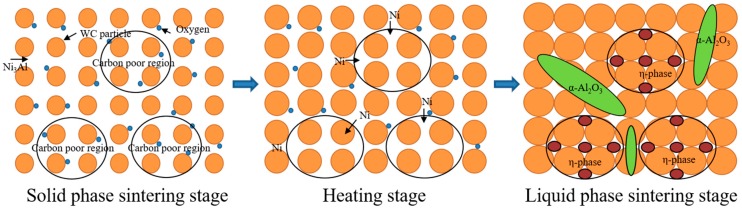
Schematic diagram for the generation of η-phase and α-Al_2_O_3_ in WC-Ni_3_Al composites milled by CH_3_CH_2_OH.

Ni_3_Al as a bulk material has good oxidation resistance, but it is inevitable that oxygen will be doped in the WC-Ni_3_Al composites prepared by the traditional powder metallurgy method. The oxygen in the organic solvent CH_3_CH_2_OH is easily adsorbed in the mixed powder. The oxygen content of the ball-milling powder is obviously higher than that of the original powder, which indicates that the adsorption of oxygen on the surface of the powder increases with the increase of the specific surface area of the wet-milling powder. In the samples using CH_3_CH_2_OH as ball-milling suspension, the main relationship between oxygen content and microstructure is the formation of α-Al_2_O_3_ and the change of Ni_3_Al composition. Figure 8 shows a schematic diagram of η-phase and α-Al_2_O_3_ formation in CH_3_CH_2_OH ball-milled WC-Ni_3_Al composites. Some oxides and carbides are produced by sintering WC-10Ni_3_Al mixed powders in the presence of a large amount of oxygen. The decrease of oxygen content after sintering and the decrease of vacuum level during sintering proved that the formation of CO and the loss of carbon lead to a reduction of carbon in the matrix. In the process of liquid phase sintering, the formation of α-Al_2_O_3_ inevitably leads to changes in the Ni_3_Al composition. On the other hand, the diffusion of Ni to the carbon poor regions and the formation of a carbon-poor phase maintain the carbon balance in the material. The η-phase (Ni_3_W_3_C) and α-Al_2_O_3_ in CH_3_CH_2_OH ball-milled WC-Ni_3_Al composites were confirmed by TEM and EDS analysis.

In addition, in WC-Co system, the Co_3_W_3_C produced by lack of carbon is the main factor to restrain WC grain growth, so the controlling factor of WC grain growth rate depends on carbon content [22]. When the carbon content is low and the η-phase is formed, the self-diffusion of carbon is the controlling factor for the rate of dissolution and precipitation of W and C into WC. In the same way, the formation of the η-phase (Ni_3_W_3_C) in WC-Ni_3_Al composites is also the reason for inhibiting grain growth. As shown in Figure 2a,b, the grain size of CH_3_CH_2_OH ball-milled samples is fine and uniform, while there is some abnormal grain growth in the C_6_H_12_ ball-milled samples. These results show that control of oxygen content is an important factor in the preparation of WC-Ni_3_Al composites with controllable structure.

**Figure 9:**
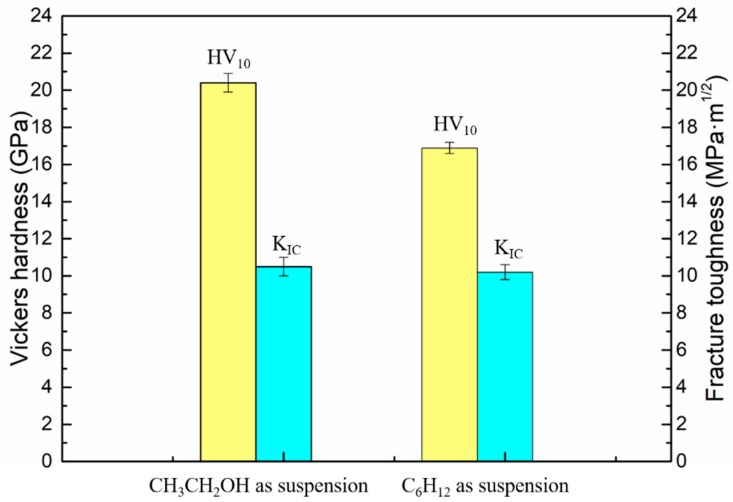
Hardness and fracture toughness of the sintered WC-10Ni_3_Al composites using different ball-milling suspensions.

Figure 9 shows the hardness and fracture toughness of WC-Ni_3_Al composites using two different ball-milling suspensions. The hardness of CH_3_CH_2_OH ball-milled samples (20.40 GPa) is higher than that of C_6_H_12_ ball-milled samples (16.89 GPa). This is because the WC-Ni_3_Al composite conforms to the Hall-Petch relationship in the range of submicron grains, and the decrease in grain size leads to the increase of hardness. It is interesting, however, that there is little difference in fracture toughness, 10.5 Mpa·m^1/2^, and 10.2 Mpa·m^1/2^, respectively.

**Figure 10:**
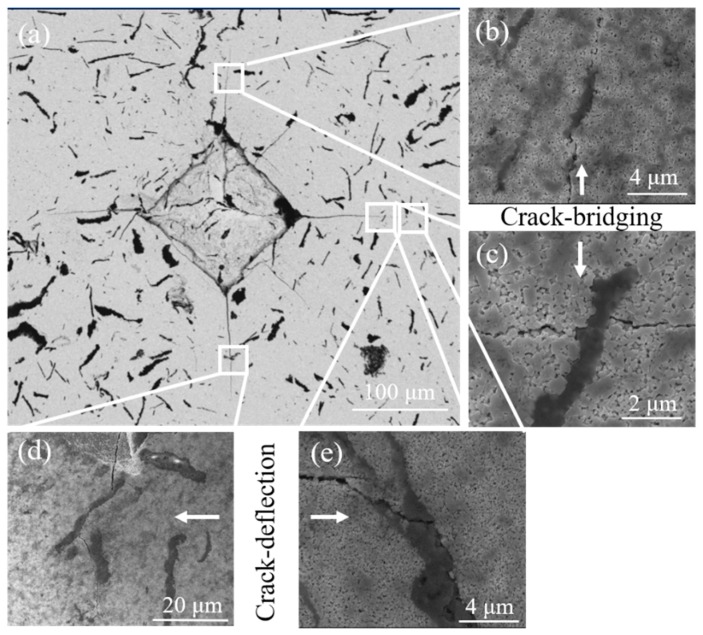
SEM images of indentation cracks observed in the sintered WC-10Ni_3_Al composites using CH_3_CH_2_OH. (**a**) Vickers indentation; (**b**,**c**) crack-bridging; (**d**,**e**) crack-deflection.

**Figure 11:**
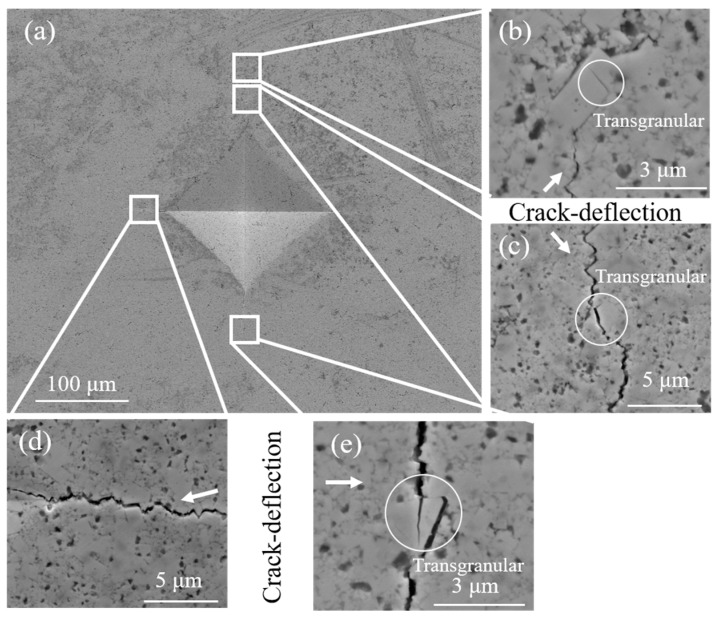
SEM images of indentation cracks observed in the sintered WC-10Ni_3_Al composites using C_6_H_12_. (**a**) Vickers indentation; (**b**–**e**) crack-deflection with transgranular.

In order to understand the toughening mechanism of WC-Ni_3_Al composites using two different ball-milling suspensions, the Vickers hardness indentation crack on the surface of WC-Ni_3_Al composites were observed. Generally speaking, the crack propagation path in WC-based cemented carbide can be divided into intergranular and transgranular. When the grain size is small, the intergranular fracture is formed at the WC/WC grain boundary and the WC/binder interface. When the grain size is large, there will be a transgranular fracture in the large grains. As shown in Figure 10, it can be found that intergranular fracture is the dominant mechanism in CH_3_CH_2_OH ball-milled samples with small grain size. It should be noted that α-Al_2_O_3_ can deflect and bridge the crack growth and increase the resistance to crack growth. This explains why the grain size of CH_3_CH_2_OH ball-milled samples is smaller, but the toughness does not decrease. Figure 11 is the micrograph of crack propagation in C_6_H_12_ ball-milled sample. It can be seen that the abnormally grown plate-like grains have the same deflection effect on the crack, even when the crack passes through the plate-like grains. When large grains are encountered, deflection and transmigration are more likely to occur simultaneously, as shown in Figure 11e. It should be noted that more energy is needed to form a transgranular crack, which hinders crack propagation. Therefore, the toughness of C_6_H_12_ ball-milled samples is better due to the existence of plate-like crystals.

## 4. Conclusions

(1) WC-Ni_3_Al composites were consolidated using SPS, and a small amount of α-Al_2_O_3_ was formed in the samples which used ethanol (CH_3_CH_2_OH) as a ball-milling suspension. The oxygen mainly comes from the adsorption and dissolution of oxygen atoms in organic solvent during ball-milling.
(2) The WC-Ni_3_Al composite powders milled with ethanol (CH_3_CH_2_OH) contain a large amount of oxygen, one part of which forms α-Al_2_O_3_, the other part forms CO gas, which leads to carbon deficiency in the WC matrix, and the formation of η-phase (Ni_3_W_3_C) maintains the carbon balance in the materials.
(3) In the WC-Ni_3_Al composites milled with ethanol (CH_3_CH_2_OH), the formation of α-Al_2_O_3_ enhances its fracture toughness due to crack deflection and bridging, and the formation of η-phase (Ni_3_W_3_C) inhibits the grain growth and increases its hardness. The hardness can be increased without loss of toughness.
(4) In the WC-Ni_3_Al composites milled with cyclohexane (C_6_H_12_), the abnormal grain growth leads to a decrease in hardness, but the fracture toughness is maintained due to crack deflection and crack bridging.

## Figures and Tables

**Table 1:** Representative elemental distributions for the WC-10Ni_3_Al specimen made using CH_3_CH_2_OH analyzed by TEM-EDS system (spot size 100 nm).

Spot	Elements (at.%)					
	W	Ni	Al	O	C	Cr
1	42.46	2.01	—	1.61	53.92	—
2	—	—	30.97	54.03	13.56	1.44
3	34.83	34.55	12.34	17.02	0.73	0.53
4	3.32	73.59	19.65	—	0.43	3.01
5	56.60	—	—	—	43.40	—
6	3.49	67.39	25.26	—	0.03	3.83
7	31.12	51.03	7.06	6.37	1.93	2.49
8	1.34	0.02	31.13	65.40	—	2.11
